# Combined GC–MS and RNA-Seq Identification of the Role of the ABC Gene Family in the Formation of Mango Flavor Compounds

**DOI:** 10.3390/plants14182915

**Published:** 2025-09-19

**Authors:** Yibo Bai, Songlin Yang, Dairui Hou, Hanqing Cong, Huapeng Sun, Rongxiang Wang, Xiaona Fu, Fei Qiao

**Affiliations:** Tropical Crops Genetic Resources Institute, Chinese Academy of Tropical Agricultural Sciences, National Key Laboratory for Tropical Crop Breeding, Key Laboratory of Crop Gene Resources and Germplasm Enhancement in Southern China, Ministry of Agriculture and Rual Affairs, Key Laboratory of Tropical Crops Germplasm Resources Genetic Improvement and Innovation of Hainan Province, Haikou 571101, China; ybbai@catas.com (Y.B.); lsyangyn@163.com (S.Y.); 15246872753@163.com (D.H.); hanqing.cong@catas.cn (H.C.); huapeng_sun@catas.cn (H.S.); wrxiang1972@163.com (R.W.); 13700479319@163.com (X.F.)

**Keywords:** *Mangifera indica*, ATP-binding cassette (ABC) transporters, volatile substances

## Abstract

**Background:** Mango is a tropical fruit that is deeply loved by consumers due to its unique flavor and taste. Different mango varieties have unique aromas, and the volatile components of mango are an important part of determining mango flavor. ATP-binding cassette (ABC) transporters are important in transporting plant volatile components. Although ABC transporters have been extensively studied in other species, little is known about the evolutionary characteristics and biological functions of the ABC family in mango. **Results:** In this study, a total of 119 *MiABC* genes were identified from the *Mangifera indica* genome and classified into eight subfamilies based on phylogenetic relationships. By analyzing the gene structure, subcellular localization prediction, chromosome localization, gene duplication events, and *Ka*/*Ks* ratios of *MiABC* genes, the *MiABC* gene functions were preliminarily determined. The expression profiles of *MiABC* genes at different stages of mango fruit harvesting indicate that *MiABC* genes are involved in the transport of volatile substances in mango fruit. The prediction of the transmembrane structure indicates that the *MiABC* genes have multiple transmembrane domains, and subcellular localization results show that the *MiABC* genes are mainly located on the cell membrane. **Conclusions:** In summary, this study conducted a comprehensive analysis of the ABC gene family in mango, laying an important theoretical foundation for the analysis of the transport process of volatile compounds in mango.

## 1. Introduction

Mango (*Mangifera indica* L.), a tropical evergreen tree belonging to the genus Mangifera in the Anacardiaceae family [1], represents one of the world’s major fruit crops and ranks fourth in global production [2]. Valued for its excellent taste, aroma, color, and nutritional quality, mango pulp is widely used as a raw material in beverages and various processed products [3]. The fruit can be consumed fresh or processed into forms such as mango sauce, dried slices, and juice, with its unique flavor profile being a key factor driving consumer preference [4]. Studies have identified 145 volatile compounds associated with mango aroma, including monoterpenes, sesquiterpenes, phenolics, lactones, and volatile fatty acids, among which terpenoids constitute the most significant group [5,6]. Furthermore, the composition of flavor substances varies considerably among mango cultivars, with three major aroma types characterized by terpineol, carlesene, and myrcene [7].

As a secondary metabolite, terpenoids exist in plants and play an important role in adapting to adverse environments, resisting pathogen invasion, and regulating metabolism [8]. Terpenes are mainly produced and accumulated in living organisms. The generated terpenoids need to be transported out of cells or tissues, which typically involves specific transport proteins. The transport proteins on the plant cell membrane are responsible for transporting terpenoids from synthesis sites to other required sites, such as leaves, flowers, and fruits [9]. After terpenoids reach the target site, they may further modify or accumulate to form compounds with specific biological activities, thereby achieving functions such as aroma component synthesis and defense mechanisms [10].

The production and translocation of mango flavor compounds require the involvement of multiple transporters [11]. Among them, ABC transporters constitute a class of membrane channel proteins that facilitate the movement of substances across cells. They contain highly conserved nucleotide-binding domains (NBDs) and transmembrane domains (TMDs), and utilize energy from ATP hydrolysis to transport a wide range of substrates across membranes [12]. They can utilize the energy generated by ATP hydrolysis to transfer various substrates bound to them into or out of the cell membrane. According to the structure of the ABC gene, it can be divided into eight subfamilies. ABCA has been reported to be involved in cellular lipid transport, while members of the ABCB subfamily can participate in the influx transport of auxin in roots. Members of the ABCD subfamily have been found to play a role in peroxisome transport. The ABCF subfamily proteins have been found to be involved in stress-related control. ABCC and ABCG proteins participate in membrane transport of secondary metabolites in plants [13]. Through analysis of ABC transporters in tobacco, the results show that NpABC1 can participate in the transport of diterpenoid lycorine and lycorine lactone in fungi [14], and NtPDR1 has been shown to participate in diterpenoid transport [15]. In the study of Chinese cabbage, it was found that the ABCG subfamily gene ABCG18 can affect the transport of β-caryophyllene in Chinese cabbage, leading to differences in flavor among different varieties of cabbage [16]. ABCB subfamily gene ABCB19 can transport brassinosteroids both in vitro and in plant cells [17]. ABCG subfamily genes PbABCG1 and PbABCG2 play important roles in the transport and emission of monoterpenes in the fragrance of Phalaenopsis flowers [18]. However, there are currently no reports on the relationship between mango flavor and *MiABC* genes.

In this study, we identified ABC genes associated with mango flavor compounds based on transcriptomics and metabolomics. Using the red ivory genome as a reference genome, a total of 119 ABC genes were identified in mango. By combining evolutionary tree analysis with sequence features, the ABC family members in ‘Red Ivory’ were divided into eight subfamilies. The subcellular localization prediction results indicate that most ABC genes are expressed on the cell membrane. By calculating the duplication events of the *MiABC* genes, it was found that tandem duplication events and proximal duplication events play an important role in the expansion of members of the ABC gene family in mango. The *Ka*/*Ks* ratio results show that the *Ka*/*Ks* ratio is mainly concentrated between 0.4 and 0.6, indicating that the mango ABC genes underwent purification selection during the expansion process. These results of transcriptome and metabolome analysis indicated that the ABC genes in mango were mainly expressed on days 1, 4, and 6 of the ‘Red Ivory’ mango. WGCNA analysis indicates that there was a positive regulatory relationship between eight *MiABC* genes and nine metabolites. The results of predicting the transmembrane structure of six *MiABC* genes showed that there were multiple transmembrane domains in *MiABC* genes, which were mainly expressed on plant cell membranes. These results indicate that *MiABC* genes play an important role in the transportation of volatile substances in mango.

## 2. Materials and Methods

### 2.1. Identification of Members of the Mango ABC Gene Family

The reference genome of the ‘Red Ivory’ mango was downloaded from the National Genomics Data Center (https://ngdc.cncb.ac.cn accessed on 29 August 2025) to identify the *MiABC* genes. These domains of the ABC-transporter domain (PF00005), the CYT domain (PF01458), the ABC-2 transporter domain (PF01061), and the ABC transporter transmembrane region domain (PF00664) were downloaded from the Pfam database (http://pfam.xfam.org accessed on 29 August 2025). The Hidden Markov Model (HMM) program was used for Hmmsearch to screen *ABC* genes in ‘Red Ivory’ mango based on the threshold of E < 1 × 10^−4^. The SMART database and Pfam database were used to identify candidate ABC genes in mango.

### 2.2. Construction of Phylogenetic Trees

The rice genome was downloaded from the RAP-DB database (http://rapdb.dna.affrc.go.jp/ accessed on 29 August 2025) [19]. The cabbage genome was downloaded from the BRAD database (http://brassicadb.org/brad/ accessed on 29 August 2025) [20]. Multiple sequence alignment of ABC proteins in rice, cabbage, and mango were performed by ClustalW of the MEGA11 software with default parameters [21]. IQ-tree was used as the maximum likelihood method for constructing phylogenetic trees with 1000 bootstrap replications and default parameters [22].

### 2.3. Analysis of Gene Structure

The 15 conserved motifs of ABC proteins were analyzed through the MEME website (https://meme-suite.org accessed on 29 August 2025). The CDS sequences were analyzed using Gene Structure Display Server 2.0 (https://gsds.gao-lab.org accessed on 29 August 2025). The physicochemical properties of ABC proteins were identified via the Expasy website (https://web.expasy.org/protparam/ accessed on 29 August 2025).

### 2.4. Analysis of Duplication Events and Ka/Ks Ratios

The calculation of duplication events was performed by DupGen_finder (https://github.com/qiao-xin/DupGen_finder accessed on 29 August 2025) [23], The Ka, Ks, and *Ka*/*Ks* ratios were calculated by MEGA11. The values of *Ka*/*Ks* indicated the selection mode of genes during evolution. *Ka*/*Ks* < 1 indicated that genes undergo purification selection during evolution, *Ka*/*Ks* = 1 indicated neutral selection, and *Ka*/*Ks* > 1 indicated that genes undergo positive selection during evolution.

### 2.5. Analysis of Cis-Acting Element

The promoter sequence of *MiABC* genes (2000 bp upstream of the start codon) were extracted from the genome of ‘Red Ivory’ mango. The *cis*-acting elements in the promoter sequences of *ABC* genes were identified by the PlantCARE website (http://bioinformatics.psb.ugent.be/webtools/plantcare/html/ accessed on 29 August 2025).

### 2.6. WGCNA Analysis Between ABC Transporters and Volatile Compounds

We used weighted gene co-expression network analysis (WGCNA) to analyze the correlation between ABC genes and volatile substances, and calculated the characteristic gene values (ME) for each gene module. Pearson correlation analysis was used to determine the correlation between each ME module and volatile metabolite content and obtain the correlation coefficient and significance *p*-value. Modules were defined that significantly correlated with phenotype as key modules (|Correlation Coefficient| > 0.8 and *p*-value < 0.05) for further in-depth analysis.

### 2.7. Prediction and Analysis of Subcellular Localization

The subcellular localization of *MiABC* genes expression were predicted by the WoLF PSORT website (https://wolfpsort.hgc.jp accessed on 29 August 2025). The CDS sequences of candidate genes were cloned and connected to a PJ35s vector containing a GFP tag. The plasmids PJ35s::*GWHPABLA018302*, PJ35s::*GWHPABLA021327*, and PJ35s::*GWHPABLA029095* were transferred into the GV3101 strain and the agrobacteria were resuspended containing PJ35s::*GWHPABLA018302*, PJ35s::*GWHPABLA021327*, and PJ35s::*GWHPABLA029095* vectors until OD = 1.0, respectively. The 4th to 5th leaves of two-month-old tobacco were used as materials. The resuspended bacterial solution was injected into the tobacco leaves, and the subcellular localization of candidate genes was observed by laser scanning microscopy (LSM800, Zeiss, Oberkochen, Germany).

### 2.8. Prediction of Transmembrane Structure of ABC Proteins in Mango

The candidate MiABC protein sequences were extracted from the ‘Red Ivory’ protein sequence file. The TMHMM2.0 website was used to identify the transmembrane structure of candidate MiABC proteins (https://services.healthtech.dtu.dk/services/TMHMM-2.0 accessed on 29 August 2025).

### 2.9. Sample Collection, Transcriptome, GC–MS, and qRT-PCR Analysis

For transcriptome samples, ‘Red Ivory’ fruits grown for 14 weeks were selected and sampled at room temperature at 0 d, 1 d, 2 d, 3 d, 4 d, 5 d, and 6 d. Three replicates were set up for each stage, with six fruits selected for each replicate to eliminate errors between samples. The RNA prep Pure Plant Plus Kit (Polysaccharides&Polyphenolics rich) kit (Tiangen, Beijing, China) was used to extract RNA from mango fruit, an Agilent 2100 biological analyzer (Beijing, China) was used to detect the concentration and purity of RNA, and 1% agarose gel was used to detect the integrity of RNA.

For metabolomic samples, 5 g of mango flesh from each stage was weighed and placed in a headspace bottle, immersed in a 40 °C water bath, and enriched with solid-phase microextraction fibers for 30 min to collect volatile gases generated in the sample. Then, the extraction fibers were inserted into the injection port for 1 min for analysis and data collection. Quantitative analysis of volatile compounds was carried out by GC–MS. Gas chromatography was performed using an HP-5ms capillary column. Helium was used as the carrier gas at a flow rate of 1 mL/min, with a split ratio of 20:1. The temperature program was set as follows: initial temperature of 60 °C held for 1 min, increased to 120 °C at a rate of 4 °C/min, then raised to 200 °C at 5 °C/min and held for 3 min. Electron impact (EI) was used as the ionization mode, with an electron energy of 70 eV. The mass scanning range was set from 35 to 500 m/z. The injector temperature was 250 °C, the ion source temperature was 230 °C, and the quadrupole temperature was 150 °C. Data analysis was conducted using MassHunter software B.08.00, and compound identification was performed by matching spectra against the NIST14.L library.

ChamQ Universal SYBR qPCR Master Mix (Vazyme, Nanjing, China) with the QuantStudio 6 Flex real-time PCR system (ThermoFisher, Waltham, MA, USA) were used to conduct qRT-PCR experiments; the *MiTUBB* gene was used as the reference gene. Primer information is listed on Table S3. Each qRT-PCR experiment consisted of 3 biological replicates and 3 technical replicates. The 2^−ΔΔCt^ method was used to detect and calculate the relative expression levels of genes.

## 3. Results

### 3.1. Genome-Wide Identification of ABC Family Genes from Mango

To identify ABC family genes from *M. indica*, the ABC transporter domain, ABC-2 transporters domain, and ABC transporter transmembrane region domain in the genome were used as query criteria for HMMER analysis. We identified a total of 119 *MiABC* genes, each containing at least one of the aforementioned domains. Based on the analysis of the physicochemical properties of the MiABC proteins, we identified that the theoretical isoelectric points (PI) of MiABC family members mainly range from 5.53 to 9.85, with most MiABC members being alkaline. In addition, the molecular weight of the 119 MiABC family members primarily ranges from 15 to 212 KDa.

### 3.2. Phylogenetic Analysis of MiABC Genes

**Figure 1:**
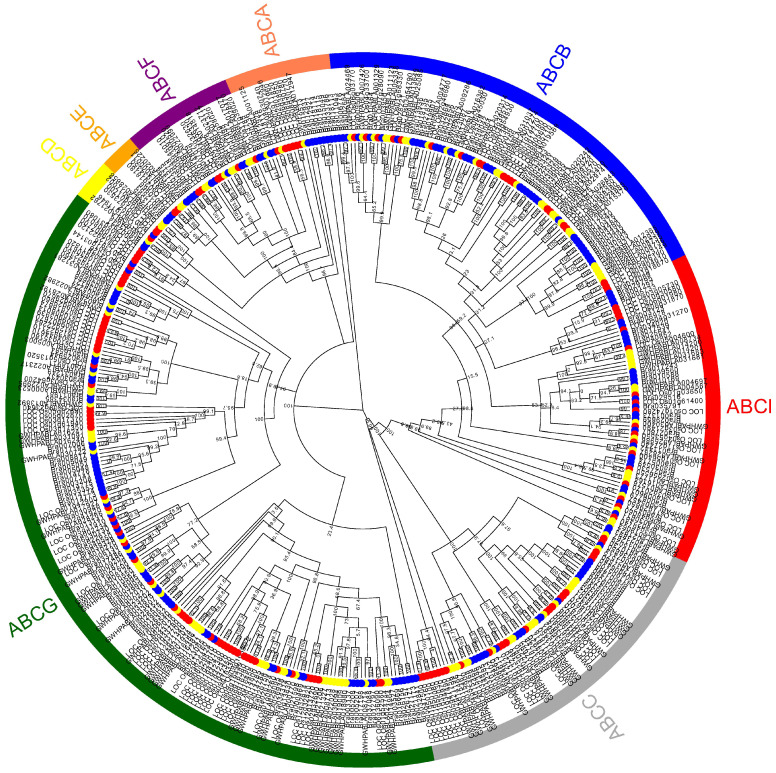
Phylogenetic tree of *ABC* genes between *Oryza sativa*, *Brassica rapa*, and *Mangifera indica*. Different colored backgrounds represent the eight subfamilies. *Oryza sativa* (red), *Brassica rapa* (blue), and *Mangifera indica* (yellow) are represented by circles of different colors.

Based on the different structural domains of the ABC gene family members in mango, combined with the reported ABC gene members in rice and cabbage, we divided the ABC gene family members in mango into eight subfamilies (Figure 1), namely ABCA subfamily, ABCB subfamily, ABCC subfamily, ABCD subfamily, ABCE subfamily, ABCF subfamily, ABCG subfamily, and ABCI subfamily. Among them, 17 ABC proteins of mango do not contain the NBD domain. Based on previous studies, the 17 ABC proteins of mango were defined as the ABCI subfamily [24]. The 42 ABC proteins of mango were defined as the ABCG subfamily. The seven ABC proteins of mango were defined as the ABCF subfamily. The three ABC proteins of mango were defined as the ABCE subfamily. The two ABC proteins of mango were defined as the ABCD subfamily. The 29 ABC proteins of mango were defined as the ABCB subfamily. The 17 ABC proteins of mango were defined as the ABCC subfamily.

### 3.3. Conserved Motif and Gene Structure Analyses of MiABC Genes

**Figure 2:**
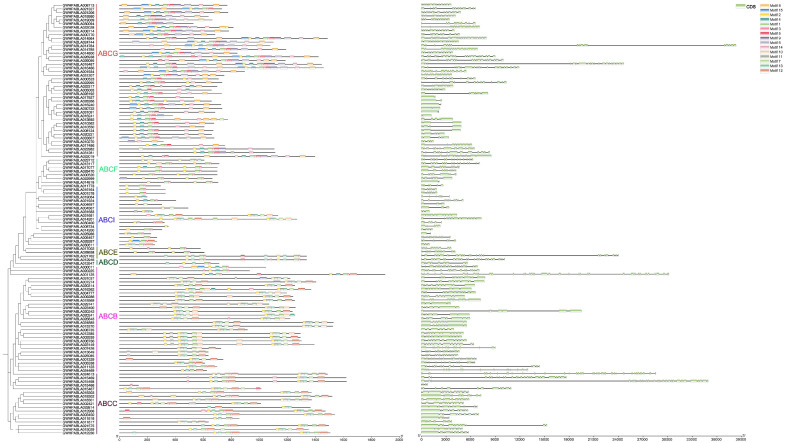
Conserved motif and gene structure analyses of ABC genes in *Mangifera indica*. The different colors on the evolutionary tree represent different subfamilies; different motifs are represented by different colors, and the light green boxes represent the number of CDS sequences.

We performed a conserved motif analysis on the amino acid sequence of MiABC, with the number of motifs set to 15. As shown in Figure 2, there are distribution differences in motifs within each subfamily. There are a large number and types of motifs in the genes of the ABCG subfamily, including 15 motifs. Motif 10 (ABC transporter B family) only exists in the ABCB subfamily. In the ABCF subfamily, is mainly included motif 1 (ATP-Binding cassette subfamily B), motif 8 (ATP-Binding cassette transporter), and motif 5 (Pleiotropic drug resistance protein 1-like isoform X1). In the ABCF subfamily is mainly included motif 1, motif 2 (P-loop NTPase-P-loop containing nucleoside triphosphate hydrolase), and motif 14 (Pleiotropic drug resistance protein 1-like isoform X1). The ABCB subfamily mainly includes four types of motifs, including motif 2,11 (P-loop NTPase-p-loop containing nucleoside triphosphate hydrolase), motif 4 (pleiotropic drug resistance protein 1-like isoform X1), motif 7 (ATP-binding cassette subfamily B), and motif 12 (ABC transporter B family). To further characterize the gene structure of the ABC family in mango, the distribution of CDS sequences was identified, with the number of CDS sequences ranging from 2 to 40, and those from the same subfamily having similar gene structures.

To further investigate the *MiABC* genes, we extracted the promoter sequence upstream of the CDS sequence of MiABC by 2000 bp for analysis of cis-acting elements. There were 14 types of cis-acting elements, including defense and stress response, light response, hormone signal elements, circadian control, endosperm expression, and stress induction elements (Figure S1). This result suggested that *MiABC* genes may be involved in the transport of substances in response to various environmental factors, thereby ensuring the normal growth and development of plants and protecting them from environmental stress.

### 3.4. Chromosomal Localization, Duplication Events, and Synteny Analysis

**Figure 3:**
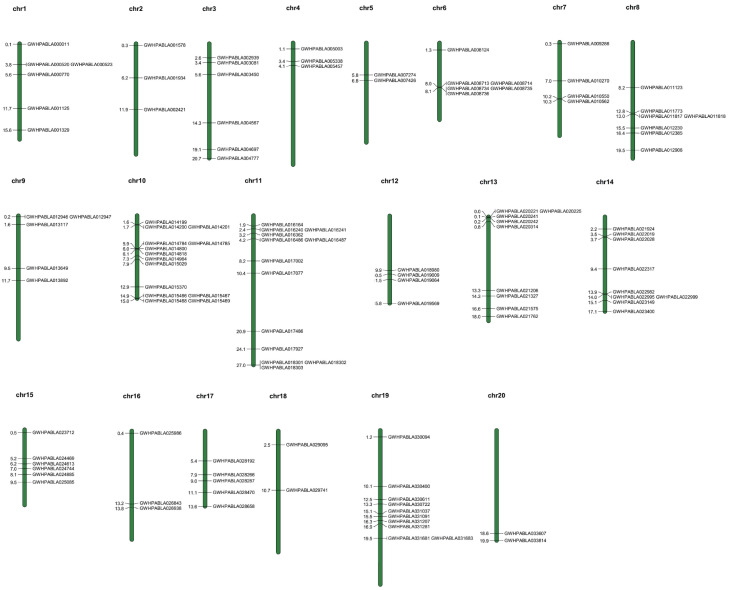
Chromosome distribution map of *MiABC* gene family. Chr1-20 represents the number of chromosomes, and the numbers on chromosomes indicate the specific locations of genes.

The chromosome localization results show that 119 *MiABC* genes were unevenly distributed on 20 chromosomes, with Chr 5 and Chr 20 having the fewest number of *MiABC* genes (2), and Chr 10 having the highest number of *MiABC* genes (14) (Figure 3). Among the 119 *MiABC* genes, we found that there were tandem duplication events between 61 *MiABC* genes and proximal duplication events between 37 *MiABC* genes. These results indicate that tandem duplication and proximal events play important roles in *MiABC* genes expansion.

**Figure 4:**
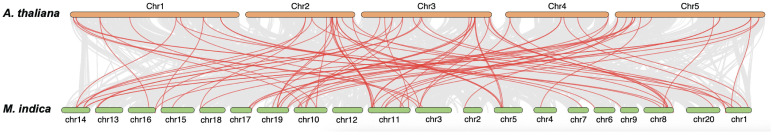
Collinearity relationships of *ABC* genes among *A. thaliana* and *M. indica*.

To further identify the orthologous genes and their evolutionary relationships of MiABCs, we compared the homology between *Arabidopsis* and mango and identified a total of 72 pairs of orthologous *MiABC* genes between *Arabidopsis* and mango (Figure 4).

**Figure 5:**
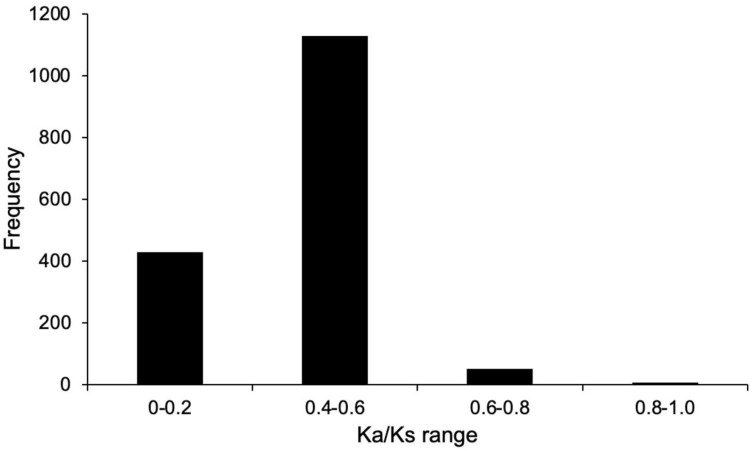
The *Ka*/*Ks* ratios of duplication events in *MiABC* genes.

In this study, we analyzed the characteristics of MiABC family members during the evolution process. The *Ka*/*Ks* values of mango ABC genes were less than 1 and mainly concentrated between 0.4 and 0.6. This result indicates that *MiABC* genes underwent significant purification selection during the evolution process (Figure 5).

### 3.5. Expression Patterns of MiABC Genes at Different Maturity Stages of Mango Fruits

**Figure 6:**
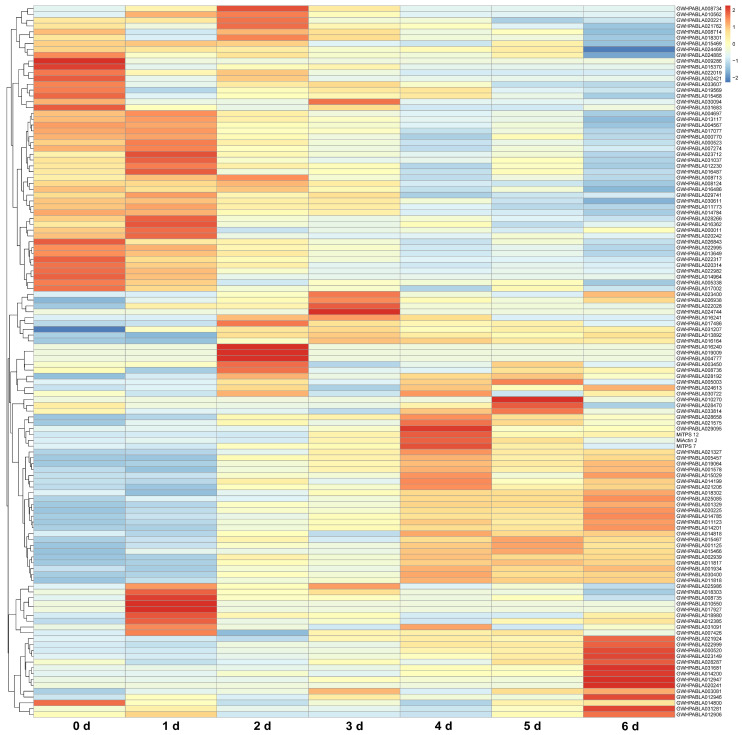
Analysis of MiABC, MiActin 2, MiTPS 7, and MiTPS 12 gene expressions at different time points after mango harvesting. The colors from blue to red represent a gradual increase in expression level, while 0 d, 1 d, 2 d, 3 d, 4 d, 5 d, and 6 d represent different days after harvesting.

We conducted transcriptome and metabolome analysis on mango fruits at different stages after harvesting. The results show that approximately one quarter of *MiABC* genes were highly expressed within 1–3 d after harvesting, and approximately one half of *MiABC* genes were highly expressed within 4–6 d. Moreover, the *MiABC* genes highly expressed within 1–3 d were different from those highly expressed within 4–6 d. These results indicate that with the extension of post-harvest time, the expression level of *MiABC* genes gradually increases, and the expression of *MiABC* genes varies at different stages after harvest (Figure 6).

**Figure 7:**
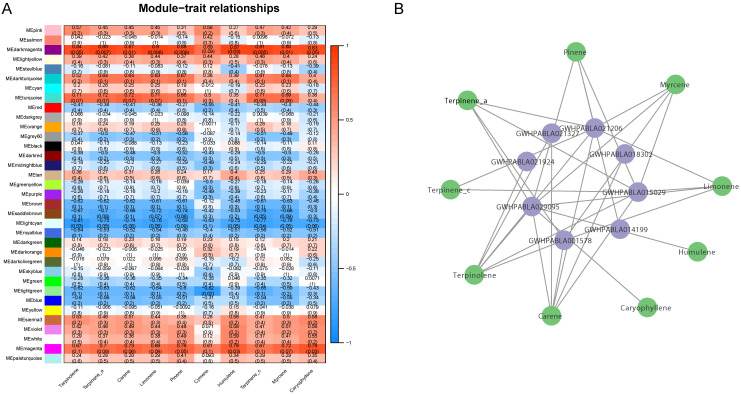
WGCNA analysis of the positive correlation between *MiABC* genes and volatile metabolites in mango. (**A**) WGCNA analysis reveals the relationship between different volatile compounds and gene modules, with colors ranging from blue to red representing correlation coefficients from low to high. (**B**) The relationship between 8 ABC genes and 9 volatile metabolites.

In order to further clarify the relationship between the expression of *MiABC* genes and volatile substances in mango, the GC–MS results show that during the ripening process of mango fruit, 10 metabolites were mainly released, including terpinene-a, terpinene-c, terpinene, carene, caryophyllene, humulene, limonene, cymene, myrcene, and pinene. WGCNA analysis divided the genes in mango into 37 modules, among which the MEdarkmagenta module showed a high positive correlation (|Correlation Coefficient| > 0.8) with nine volatile metabolites. By analyzing the genes in MEdarkangenta, it was found that there is a high positive correlation (|Correlation Coefficient| > 0.8) between eight *MiABC* genes (GWHPABLA021327, GWHPABLA021206, GWHPABLA021924, GWHPABLA029095, GWHPABLA001578, GWHPABLA014199, GWHPABLA015029, GWHPABLA018302) and nine volatile metabolites (see Figure 7).

**Figure 8:**
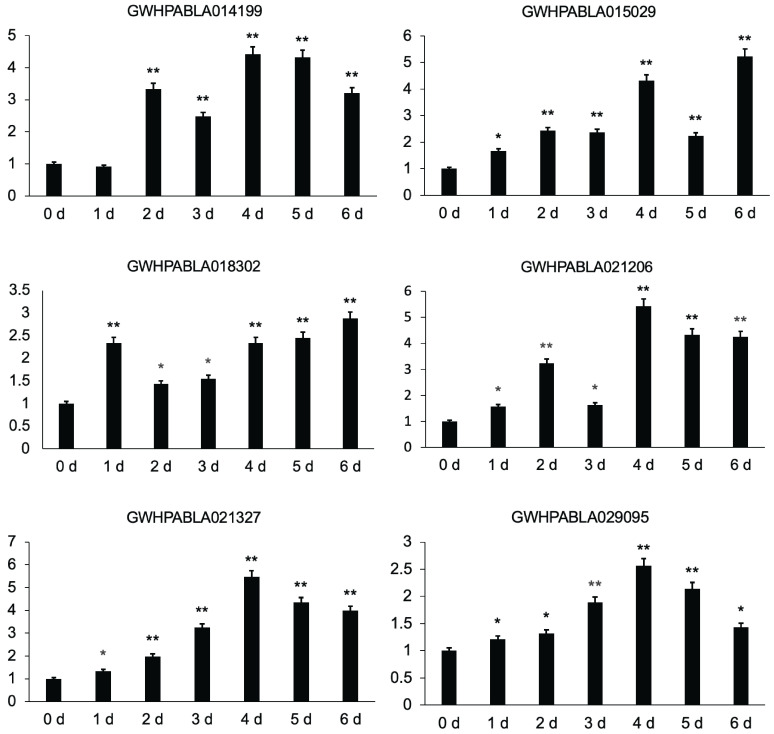
qRT-PCR analysis of the expressions of *MiUGT* genes at different time points after mango harvesting (Student’s *t*-test, * *p* < 0.05 and ** *p* < 0.01).

We selected ABCC and ABCG subfamily genes (GWHPABLA021327, GWHPABLA021206, GWHPABLA029095, GWHPABLA014199, GWHPABLA015029, GWHPABLA018302) for qRT-PCR analysis, and the results show that these six genes mainly increased with time from 0 to 4 d and were highly expressed from 4 to 6 d (Figure 8). Among them, the genes of GWHPABLA014199, GWHPABLA021206, GWHPABLA021327, and GWHPABLA029095 were highly expressed at 4 d, and their expression levels slightly decreased at 5–6 d. In addition, the contents of nine volatile substances were highest at 4 d, which is similar to the expression of *MiABC* genes, indicating that *MiABC* genes play an important role in the transportation of volatile substances in mango.

### 3.6. Analysis of the Transmembrane Structure of MiABC Proteins

**Figure 9:**
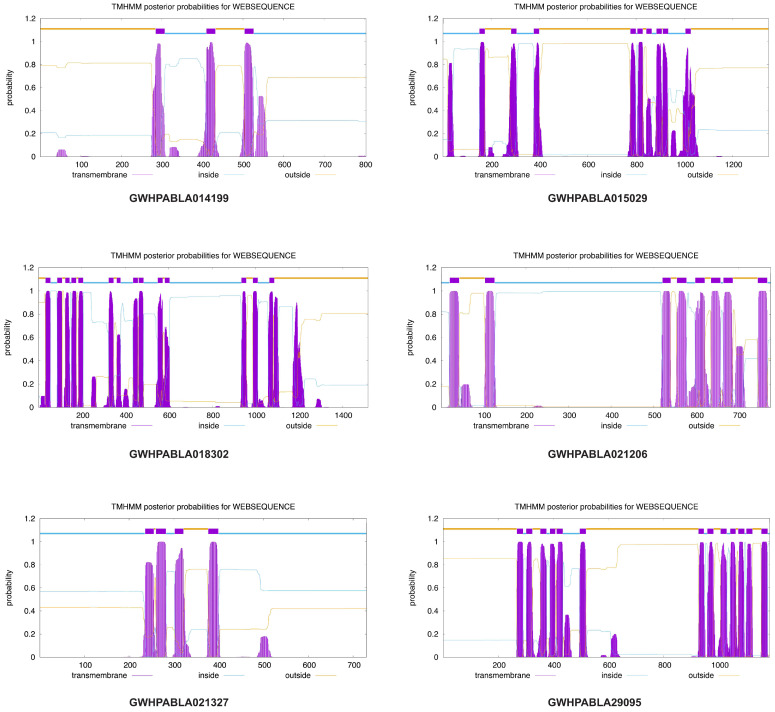
Analysis of the transmembrane domains of *MiABC* genes in mango.

The expansion analysis of ABC proteins shows that ABCB, ABCC, and ABCG are the largest subfamilies involved in the expansion process during evolution and can participate in the transport of secondary metabolites and plant hormones [25]. To further validate the critical role of MiABC proteins in the transportation of volatile compounds in mango, we predicted the transmembrane structures of six proteins (GWHPABLA021327, GWHPABLA021206, GWHPABLA029095, GWHPABLA014199, GWHPABLA015029, GWHPABLA018302). They belong to the ABCC and ABCG subfamilies, respectively. The THHMM2.0 prediction results indicate that the protein of GWHPABLA014199 had six transmembrane domains, mainly concentrated in the 300–600 amino acid sequence. The protein of GWHPABLA015029 had nine transmembrane domains, mainly concentrated between the 0–400 amino acid sequence and the 800–1000 amino acid sequence. The transmembrane domain of the GWHPABLA018302 protein was mainly concentrated between the 0–600 amino acid sequence and 900–1200 amino acid sequence. The transmembrane domain of GWHPABLA021206 protein was mainly concentrated between the 0–100 amino acid sequence and 500–800 amino acid sequence. The protein of GWHPABLA021327 had three transmembrane domains, mainly concentrated in the 200–400 amino acid sequences. The transmembrane domain of GWHPABLA29095 protein was mainly concentrated between the 200–600 amino acid sequence and 900–1200 amino acid sequence (Figure 9).

### 3.7. Subcellular Localization of MiABC Genes

**Figure 10:**
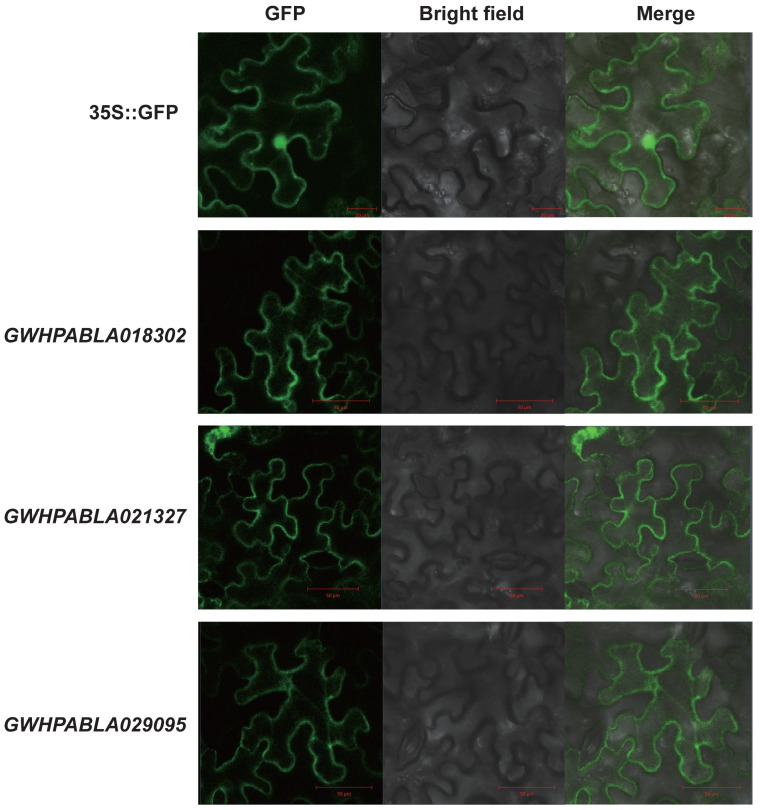
Subcellular localization analysis of *MiABC* genes. The three columns represent the bright field, dark field, and merge field, respectively. Different gene names represent the subcellular localization of different genes.

To further validate the function of *MiABC* genes, we selected three genes (GWHPABLA018302, GWHPABLA021327, GWHPABLA029095) from the ABCC and ABCG subfamilies for subcellular localization analysis. Subcellular localization experiments under laser scanning microscopy showed that the GFP signals of these three genes (GWHPABLA018302, GWHPABLA021327, GWHPABLA029095) were mainly detected on the cell membrane (Figure 10). This result indicates that these three genes were mainly expressed on the cell membrane, laying the foundation for their transport function with ABC transporters.

## 4. Discussion

Flavor, which arises from the combination of aroma, taste, and texture, is a key determinant of the market value of fruits. Volatile compounds play a central role in flavor, providing distinctive sensory characteristics [26]. As a tropical fruit renowned for its unique flavor, mango contains a high abundance of volatile compounds. The types and proportions of these aromatic volatiles significantly influence the overall sensory quality of the fruit [27,28].

As one of the oldest and largest protein families in nature, ABC transporters facilitate the transport of essential molecules and play a crucial role in important biological processes in plants [29]. These ATP-binding cassette (ABC) transporters are responsible for the transmembrane movement of diverse substrates, including amino acids, vitamins, sugars, lipids, metal ions, and secondary metabolites [30]. Currently, ABC transporters have been identified in multiple species, with 129 AtABC genes in Arabidopsis [31], 123 OsABC genes in rice [32], 117 PdABC genes in almonds [33], and 115 FvABC genes in woodland strawberry [34]. In our study, we identified 119 *MiABC* genes in the ‘Red Ivory’ mango genome, which were classified into eight subfamilies using a phylogenetic tree. Our results are consistent with previous research [35]. Analysis of their gene structures and physicochemical properties showed that *MiABC* genes from different subfamilies have similar motif structures and exon distributions. This result suggests that the same subfamily of *MiABC* gene members may exercise similar functions [24]. As a membrane protein superfamily, ATP binding cassette (ABC) transporters are responsible for ATP-driven transport of many substrates on the membrane [36]. The subcellular localization prediction results indicate that the majority of *MiABC* genes are located on the plasma membrane. This also lays the foundation for further research on the transport function of MiABC proteins [37].

Similar to the ABC gene family in other fruit trees, *MiABC* gene members were unevenly distributed on 20 chromosomes due to different duplication events [38,39]. The duplication event was the main mode of gene family expansion [40]. In the analysis of duplication events in *MiABC* genes, it was found that proximal duplication and tandem duplication were the main duplication modes of MiABC genes, with 61 *MiABC* genes experiencing tandem duplication. Similarly, tandem duplication dominates in the expansion of the ABC gene family in Chinese cabbage [35]. These results indicate that tandem duplication events were crucial for the rapid expansion and evolution of the *MiABC* gene family. In addition, there were 72 pairs of collinear genes between the *MiABC* gene and *AtABC* gene in mango, indicating that there may be similar functions between the *MiABC* genes and *AtABC* genes. The ABC genes were involved in various growth, development, and metabolic processes in plants [41]. ABCG29 can participate in the transport of coumarin to plant cell walls [42]. ABCG1 plays an important role in the transport of fatty acids and fatty alcohols [43]. These results suggest that the *MiABC* gene may be involved in the transport of secondary metabolites in plants. The *Ka*/*Ks* ratio represents the selection mode of duplicated genes [44]. Similar to the research results on PbABC genes in almonds [33], the *Ka*/*Ks* ratios of duplicated *MiABC* genes were less than 1 and mainly concentrated between 0.4 and 0.6, indicating that the mango ABC genes had been subjected to strong purification selection pressure during evolution.

ABC transporters play an important role in plant growth and development. Previous studies have shown that OsMRP15 and VvABCC1 were involved in the transport of anthocyanins in rice [45] and grapes [46], respectively. The CmABCC10 transporter can alter the distribution and content of auxins and flavonoids during plant growth and development through its transport activity [47]. ABC transporters have also been reported to be involved in the release process of plant volatile organic compounds. PhABCG1 controls the biosynthesis of VOCs in petunias, thereby regulating the release of their aroma [48]. PhABCG1 mediates the release of floral compounds from Clarkia breweri [49]. Through analysis of 224 resequencing data of mango, it was found that the transcription level of genes encoding ABC transporters gradually increases during the ripening process of mango fruits, and special SNPs related to β-laurene and D-limonene content were identified. These results laid the foundation for investigating the role of ABC transporters in mango flavor release processes [50]. By combining transcriptome and metabolome data, we further analyzed the relationship between *MiABC* genes and volatile compounds in mango. The transcriptome and qRT-PCR analysis results show that *MiABC* genes were mainly highly expressed after the fourth day of mango harvesting. Similarly, the content of terpenes in mango fruits was the highest on the fourth day after harvest. WGCNA analysis showed that there was a high correlation between eight *MiABC* genes and nine volatile compounds. Due to the reported involvement of ABCC and ABCG subfamilies in the transport of plant secondary metabolites, we mainly predicted the transmembrane structures of ABCC (GWHPABLA014199, GWHPABLA015029, GWHPABLA018302) and ABCG (GWHPABLA021327, GWHPABLA021206, GWHPABLA029095) subfamily members in mango, which were consistent with the characteristics of ABC gene family members. By selecting ABCC subfamily member GWHPABLA018302 and ABCG subfamily members GWHPABLA021327 and GWHPABLA029095 for subcellular localization analysis, it was found that *MiABC* genes were mainly expressed on the plasma membrane. These results indicate that *MiABC* genes, like ABC genes in other species, can participate in the transport of volatile compounds related to mango aroma as membrane proteins [42,45]. This study laid a theoretical foundation for further in-depth research on the function of *MiABC* genes.

This study only used one mango variety, ‘Red Ivory’, so the generalizability of the research conclusion to other varieties with different genetic backgrounds still needs further verification. Secondly, our observation period is limited to post-harvest and does not cover the complete shelf life and decay process of the fruit. In future research, we will use multi-variety comparative analysis, and extend the observation time and combine functional verification methods such as genetic modification or in vitro biochemical experiments to further elucidate the relationship between ABC transporter proteins and mango volatile substance release.

## 5. Conclusions

Mango is deeply loved by consumers due to its unique flavor, and its tropical aroma is mainly composed of various volatile substances. However, little is currently known about the transport process of volatile substances in mango. In this study, we focused on whole gene family identification of ABC transporters and identified 119 *MiABC* genes. The phylogenetic tree divided MiABC family members into eight subfamilies. By analyzing the structure and evolutionary mode of *MiABC* genes, it was found that *MiABC* genes can participate in the transport process of various biological functions. Combining transcriptome and metabolome analyses, it was found that during the post-ripening process of mango, the *MiABC* genes act as membrane proteins with multiple transmembrane domains and exhibit a high positive correlation with various volatile substances. These research results provide new insights into the transport of mango aroma compounds by ABC transporters for subsequent studies.

## Data Availability

Data are contained within the article.

## Supplementary Materials

The following supporting information can be downloaded at: https://www.mdpi.com/article/10.3390/plants14182915/s1, Figure S1. Cis-acting elements analysis of *MiABC* genes; Figure S2. Changes in volatile metabolite content during different post-harvest periods; Table S1. Subcellular localization prediction of ABC genes in mango; Table S2. Duplication events of ABC genes in mango; Table S3. Primer sequences used to detect the expression of *MiABC* genes; Table S4. Cis-element analysis of promoter sequences of 8 candidate genes.
