# Supplementary material for: Combined GC–MS and RNA-Seq Identification of the Role of the ABC Gene Family in the Formation of Mango Flavor Compounds
Source: Plants (Basel). 2025 Sep 19;14(18):2915. doi: 10.3390/plants14182915 (PMC12473896; doi:10.3390/plants14182915)
Supplement: Supplementary file 1 [file plants-14-02915-s001.zip › Supplementary Figure.pdf]

Supplementary

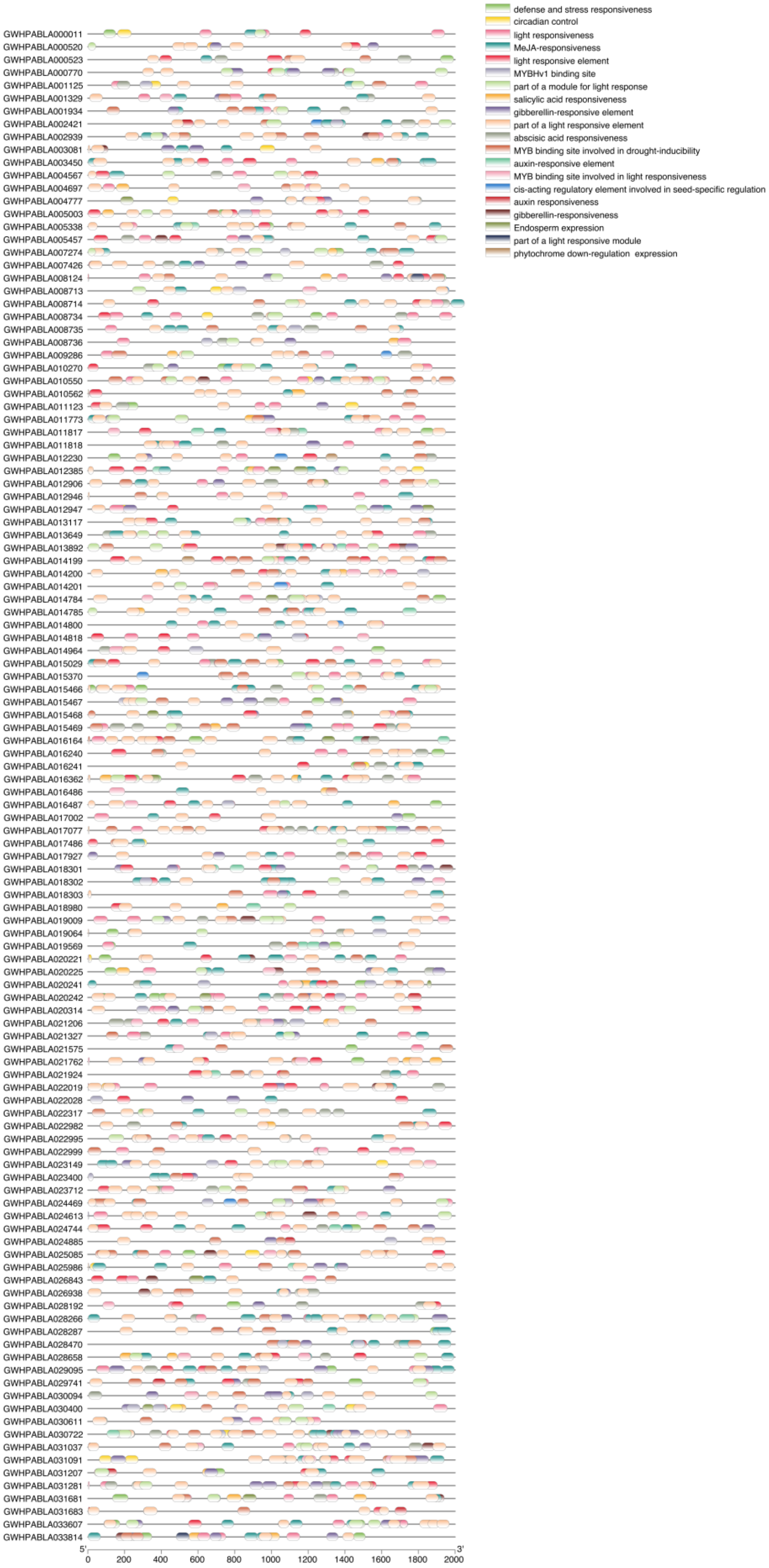

Figure S1. *Cis*-acting elements analysis of *MiABC* genes.

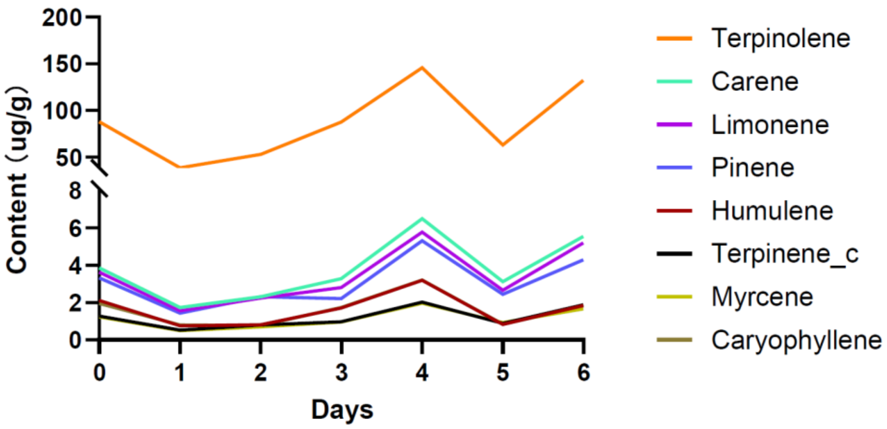

Figure S2. Changes in volatile metabolite content during different post-harvest periods.
